# Physiological responses of Holstein calves to heat stress and dietary supplementation with a postbiotic from *Aspergillus oryzae*

**DOI:** 10.1038/s41598-022-05505-3

**Published:** 2022-01-28

**Authors:** A. G. Ríus, J. D. Kaufman, M. M. Li, M. D. Hanigan, I. R. Ipharraguerre

**Affiliations:** 1 Department of Animal Science, University of Tennessee, 2506 River Drive, 235 Brehm Animal Science Building, Knoxville, TN 37996 USA; 2 Department of Dairy Science, Virginia Tech, Blacksburg, VA 24061 USA; 3 Institute of Human Nutrition and Food Science, University of Kiel, Kiel, Germany

**Keywords:** Metabolism, Gastrointestinal models, Metabolomics

## Abstract

Increased ambient temperature causes heat stress in mammals, which affects physiological and molecular functions. We have recently reported that the dietary administration of a postbiotic from *Aspergillus oryzae* (AO) improves tolerance to heat stress in fruit flies and cattle. Furthermore, heat-induced gut dysfunction and systemic inflammation have been ameliorated in part by nutritional interventions. The objective of this study was to characterize the phenotypic response of growing calves to heat stress compared to thermoneutral ad libitum fed and thermoneutral feed-restricted counterparts and examining the physiologic alterations associated with the administration of the AO postbiotic to heat-stressed calves with emphasis on intestinal permeability. In this report, we expand previous work by first demonstrating that heat stress reduced partial energetic efficiency of growth in control (45%) but not in AO-fed calves (62%) compared to thermoneutral animals (66%). While heat stress increased 20% the permeability of the intestine, AO postbiotic and thermoneutral treatments did not affect this variable. In addition, AO postbiotic reduced fecal water content relative to thermoneutral and heat stress treatments. Heat stress increased plasma concentrations of serum amyloid A, haptoglobin and lipocalin-2, and administration of AO postbiotic did not ameliorate this effect. In summary, our findings indicated that heat stress led to reduced nutrient-use efficiency and increased systemic inflammation. Results suggest that the AO postbiotic improved energy-use efficiency, water absorption, and the intestinal permeability in heat stress-mediated increase in gut permeability but did not reduce heat stress-mediated rise in markers of systemic inflammation.

## Introduction

Global climate change increases the risk of extreme heat events and is characterized by temperatures exceeding the long-term averages of magnitude, frequency, and duration^1^, threatening human and animal health as well as the economic viability of food-producing enterprises worldwide^1–4^. This is likely to worsen because the frequency and intensity of extreme heatwave events have increased in the past decades and are likely to continue to increase in the future^4^. Mammals with intensive metabolic heat production and a relatively small surface-to-volume ratio conserve heat (Bergmann’s rule) and are particularly prone to heat stress due to limited capability for radiant heat dissipation^5^. To reduce metabolic heat production, animals typically reduce feed intake, which in the case of growing cattle may account for 8 to 10% of the amount of feed consumed otherwise^3^. In combination with lower nutrient intake, blood flow shifts towards the periphery away from the splanchnic tissues to facilitate heat dissipation in the skin^5^ resulting in a shortage of oxygen and nutrients, inflammation, and oxidative stress that ultimately increases the permeability of the intestine to lumen contents^6^.

Existing heat abatement tools such as shade, ventilation, and spray cooling are used to mitigate effects of elevated ambient temperatures on-farm. However, these methods do not restore homeostasis completely^3^. This is because the efficacy of cooling systems is influenced by several animal-related factors (e.g. genetics, hair coat, sweat gland characteristics and numbers, and metabolic heat production^7^). Therefore, heat stress is known to cause health and production-related issues despite the use of economically and environmentally costly cooling technologies on farm animals^3,8,9^.

Postbiotics are defined as a “preparation of inanimate microorganisms and/or their components that confers a health benefit on the host”^10^. In line with this definition, we have recently reported that a postbiotic derived from *Aspergillus oryzae* (AO) improves tolerance to heat stress in fruit flies (*Drosophila melanogaster*) and cattle. Emphasizing the conserved nature of such a protective response, in both species the AO postbiotic induced alterations in biomarkers of immune function and inflammation reminiscent of reduced gut permeability and entry into circulation of luminal toxins and antigens^11^. This possibility, however, remains unproven because we did not examine intestinal permeability.

Therefore, the objective of this study was to expand previous work by characterizing the phenotypic response of growing Holstein bull calves to heat stress in comparison with thermoneutral feed-restricted and ad-libitum fed controls and examining the physiologic alterations associated with the administration of the AO postbiotic to heat-stressed calves with emphasis on intestinal permeability.

## Results

### Heat stress reduced energetic efficiency of growth in control but not in AO-fed calves

Feed consumption was affected by treatments (*P* < 0.01). Intake of the HSP and TNR calves was similar, and less than for HS and TN treated calves (*P* < 0.05, Table 1). Contrary to our expectations, feed intake did not differ between HS and TN animals. At first glance, this was surprising because the immediate response of an animal to heat stress is reduced nutrient consumption as an attempt to match heat production from digestion and metabolism with its heat dissipation capabilities^12^. For this reason, the TNR treatment was included to account for the effect of the expected dissimilar nutrient consumption between heat-stressed and thermoneutral animals. In support of the known detrimental effects of hyperthermia on animal productivity, partial energetic efficiency declined (*P* < 0.05) while feed efficiency (*P* < 0.08) and total energy efficiency (*P* < 0.09) tended to decline in HS compared with TN and TNR calves. This heat-induced deterioration of the efficiency of converting energy and feed into growth were partly prevented in HSP calves. Compared with TN and TNR, the HS and HSP treatments increased (*P* = 0.02) water intake on average by 3.7 L/day, but only HSP reduced (*P* = 0.04) fecal water content (73.3 vs 72.1, Table 1).

**Table 1:** The postbiotic restored partial energetic efficiency of Holstein bull calves exposed to heat stress (n = 8 per treatment).

Parameter	TN	TNR	HS	HSP^1^	SEM	*P* <
Initial BW, kg	121.7	122.0	123.1	123.3	3.49	0.98
Final BW, kg	137.4	137.4	137.0	136.0	5.60	0.99
BW gain, kg	15.4	14.4	13.2	15.2	1.55	0.59
Feed intake, kg/day^2^	4.15^a^	3.84^b^	4.1^a^	3.70^b^	0.08	0.01
Feed efficiency^3^	54.1	59.2	42.1	54.1	4.40	0.08
Total energetic eff.^3^	45.3	43.9	31.0	40.5	3.85	0.09
Partial energetic eff.^3^	66.0^a^	72.4^a^	45.9^b^	62.3^a,b^	6.05	0.04
Water intake, L/day	9.8^b^	8.8^b^	13.5^a^	12.4^a^	0.84	0.02
Daytime, L^4^	5.8^a,b^	4.9^b^	7.2^a^	7.5^a^	0.39	0.05
Nighttime, L^4^	3.9	4.0	6.2	4.6	0.83	0.32
Fecal water, %	73.3^a^	73.3^a^	72.7^ab^	72.1^b^	0.32	0.04

*TN* thermoneutral, *TNR* thermoneutral feed-restricted, *HS* heat stress, *HSP* heat stress-postbiotic, *SEM* standard error of the mean.

^a,b^Values within the same row with different superscripts denote significance differences (*P* < 0.05).

^1^AO postbiotic fed at 3 g/calf/day and mixed with milk replacer.

^2^Milk replacer + starter intake.

^3^Feed efficiency (gross BW gain/gross feed intake) × 100; Total energetic efficiency (gross energy gain/metabolizable energy intake) × 100; Partial energetic efficiency [(gross energy gain/(metabolizable energy intake − net energy maintenance)] × 100.

^4^Daytime (0500 to 1900); Nighttime (1900 to 0500).

### Heat stress increased body temperature and respiration rate

**Figure 1:**
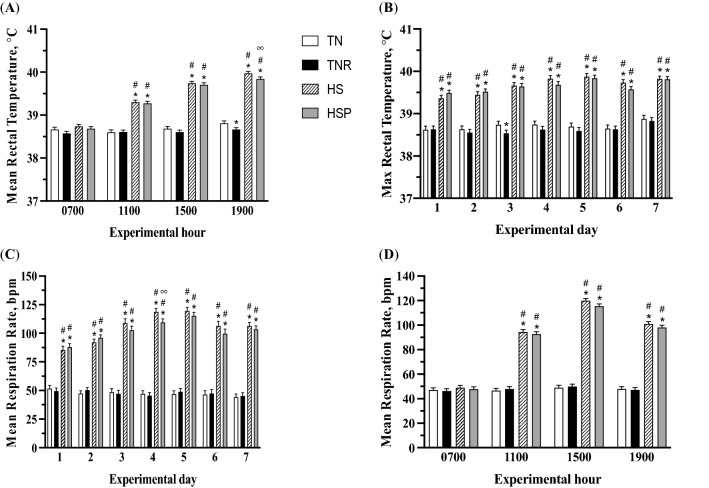
Mean (**A**) and maximum (**B**) rectal temperature of Holstein bull calves. Results denote least squares means + standard error of the mean (*TN* thermoneutral, *TNR* thermoneutral feed-restricted, *HS* heat stress, *HSP* heat stress and dietary supplementation with 3 g of AO postbiotic; n = 8 per treatment). P ≤ 0.01 denoted by *(compared against TN), ^#^(compared against TNR), or ∞ (compared against HS). (**A**) Mean rectal temperature in HSP treatment differs compared with TN, TNR, or HS; treatment by h interaction (*P* < 0.001). Mean rectal temperature in TNR treatment differs compared with TN. (**B**) Maximal rectal temperature of HS and HSP treatments differ compared with TN and TNR. Mean respiration rate (**C**) of HS and HSP treatments differ compared with TN and TNR; treatment by day interaction (*P* < 0.001). Mean respiration rate (**D**) of HS and HSP treatments differ compared with TN and TNR; treatment by day interaction (*P* < 0.001).

Surface and core body temperature and respiration rate of all calves were similar before treatment initiation (data not shown). The mean (Fig. 1A) and maximum (Fig. 1B) rectal temperature increased in HS and HSP groups compared with TN and TNR animals (treatment by h interaction; *P* < 0.01). By design, HS and HSP treatments increased mean rectal temperature on average 0.7, 1.1, and 1.2 °C, respectively at 1100, 1500, and 1700 (treatment by h interaction; *P* < 0.01; Fig. 1A). At 1900, HSP treatment decreased (*P* = 0.05) rectal temperature of calves by 0.1 °C compared with HS treatment, and TNR treatment decreased (*P* = 0.03) rectal temperature by 0.2 °C compared with TN treatment. By design, HS and HSP treatments increased maximum rectal temperature on day 1 through 7 (treatment by day interaction; *P* < 0.001; Fig. 1B).

The mean respiration rate increased on day 1 and continued this pattern thereafter in HS and HSP compared with TN and TNR calves (treatment by day interaction; *P* < 0.001; Fig. 1C). Mean respiration rate increased more in HS compared with HSP animals at day 4 (*P* ≤ 0.01). Compared with TN and TNR, HS and HSP treatments increased mean respiration rate at 1100, 1500, and 1900 h by an average of 46.4, 68.2, and 52.0 bpm, respectively (treatment by h interaction; *P* < 0.001; Fig. 1D).

**Figure 2:**
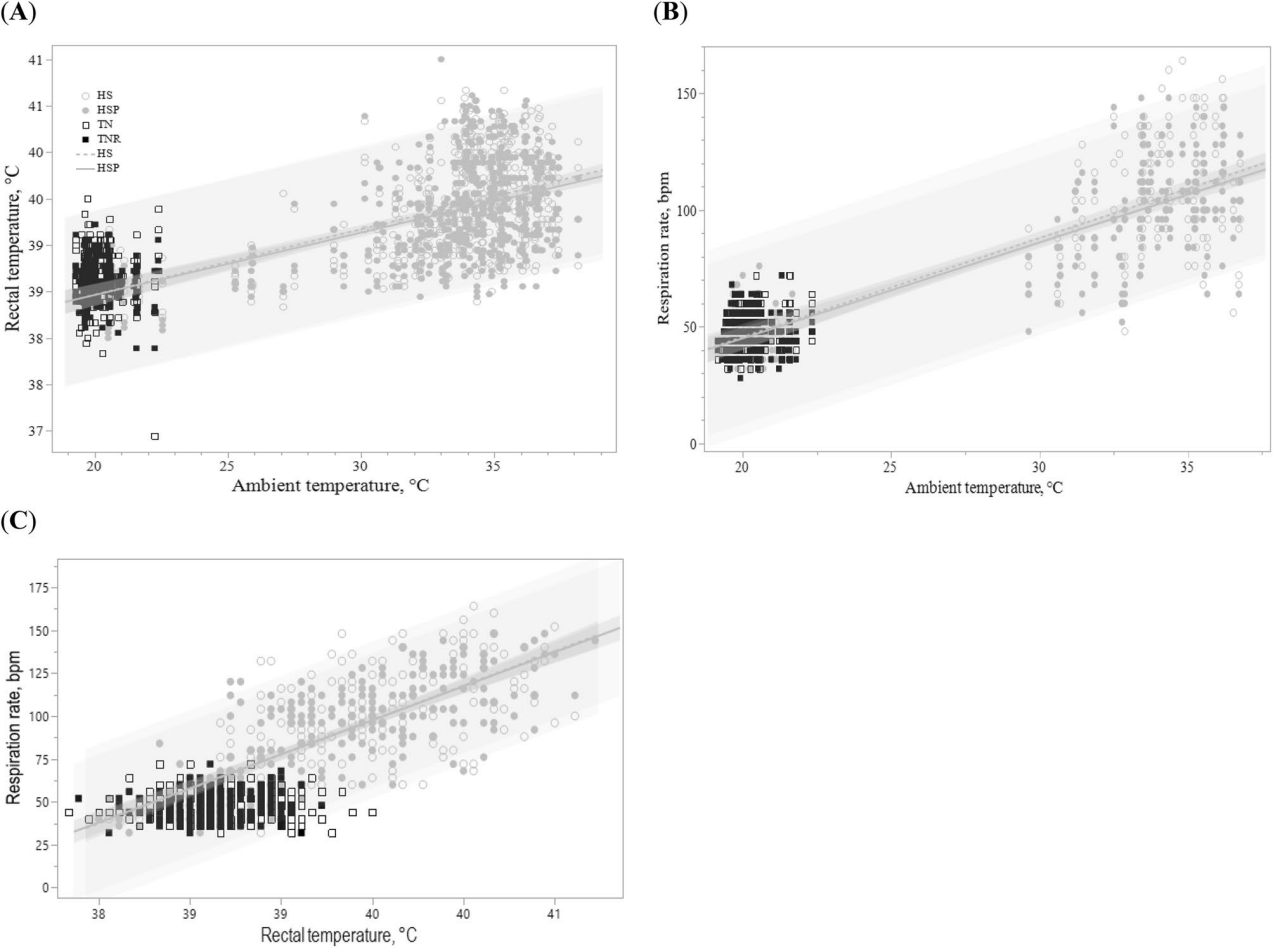
(**A**) Mean rectal temperature and (**B**) respiration rate of Holstein bull calves exposed to thermoneutral (TN), thermoneutral feed-restricted (TNR), heat stress (HS), and heat stress and dietary supplementation with 3 g of AO postbiotic (HSP) plotted against ambient temperature (n = 8 per treatment). Linear relationship is denoted in rectal temperature for HS and HSP, 37.05 + 0.07x (R^2^ = 0.30) and 37.12 + 0.06x (R^2^ = 0.28), with ambient temperature (*P* < 0.0001). (**B**) Linear relationship is shown in respiration rate for HS and HSP, 39.1 − 4.2x (R^2^ = 0.59) and 37.1 − 4.1x (R^2^ = 0.65), with ambient temperature (*P* < 0.0001). (**C**) Linear relationship is shown in respiration rate for HS and HSP, − 1485 + 40x (R^2^ = 0.52) and − 1469 + 39.6x (R^2^ = 0.59), and rectal temperature (P < 0.0001). Dark gray shaded area shows the 95% mean confidence interval and the (dashed lines) light gray represent the 95% prediction limits.

A linear relationship showed that increasing ambient temperatures explained 30.0 and 28.0% of variation in mean rectal temperatures of HS and HSP calves (*P* < 0.001; Fig. 2A). For every unit increase in ambient temperature, the mean rectal temperature increased 0.1 °C in HS and HSP calves. Similarly, increasing ambient temperatures explained 59.0 and 65.0% of variation in respiration rates of HS and HSP calves (*P* < 0.001; Fig. 2B). For every increase in ambient temperature, the respiration rate increased 4.2 and 4.1 bpm in HS and HSP calves. The increase in rectal temperatures explained 52.0 and 59.0% of the variation in respiration rates in HS and HSP animals (*P* < 0.001; Fig. 2C). A unit of rectal temperature change corresponded with 40.0 and 39.6 bpm in HS and HSP animals. Additional results of ambient temperature, relative humidity and measurements of body temperature are presented as Supplementary Information (see Supplementary Figures).

### Heat stress disrupted intestinal permeability of HS but not of HSP calves

The HS treatment increased (*P* = 0.01) the ratio of lactulose to d-mannitol compared with the TN, TNR, and HSP treatments (Table 2). Plasma d-mannitol concentrations decreased (*P* = 0.02) in the HS compared with the TN and TNR treatments. However, the HSP group registered similar lactulose: d-mannitol and d-mannitol concentration compared with TN and TNR groups.

**Table 2:** Intestinal permeability increased in HS but not in the HSP Holstein bull calves (n = 8 per treatment).

Parameter	TN	TNR	HS	HSP^1^	SEM	*P* <
Lactulose: d-mannitol	1.36^b^	1.36^b^	1.63^a^	1.41^b^	0.059	0.01
Lactulose, pmol/μL	15.9	13.0	13.1	12.0	1.6	0.42
d-mannitol, pmol/μL	10.7^a,b^	10.4^b^	6.7^c^	7.8^bc^	0.98	0.02

*TN* thermoneutral, *TNR* thermoneutral feed-restricted, *HS* heat stress, *HSP* heat stress-postbiotic, *SEM* standard error of the mean.

^a,b,c^Values within the same row with different superscripts denote significance differences (*P* < 0.05.

^1^AO postbiotic fed at 3 g/calf/day and mixed with milk replacer.

### Heat stress increased plasma concentrations of inflammatory markers

Compared with TN, HS and HSP treatments increased (*P* < 0.03) plasma concentrations of serum amyloid A by 12.8 and 17.4% (Table 3, Supplementary Figures). Compared with TN and TNR, the HSP treatment increased (*P* < 0.05) plasma concentrations of haptoglobin by 77 and 43%. The HSP increased (*P* < 0.03) plasma concentrations of lipocalin-2 by 12.3% compared with TN treatment. The TNR treatment increased (*P* < 0.03) plasma concentrations of zonulin by 47.8 and 49.1% compared with TN and HS treatments.

**Table 3:** Markers of inflammation increased in HS and HSP Holstein bull calves (n = 8 per treatment).

Parameter	TN	TNR	HS	HSP^1^	SEM	*P* <
Serum amyloid A, μg/mL	3.67^b^	3.95^a,b^	4.14^a^	4.31^a^	0.157	0.028
Haptoglobin, μg/mL	0.154^b^	0.190^b^	0.220^ab^	0.277^a^	0.0319	0.044
Lipocalin-2, pg/mL	1098^b^	1163^a,b^	1178^a,b^	1247^a^	37.5	0.025
Zonulin, ng/mL	41.2^b^	60.9^a^	31.0^b^	46.0^a,b^	7.95	0.029

*TN* thermoneutral, *TNR* thermoneutral feed-restricted, *HS* heat stress, *HSP* heat stress-postbiotic, *SEM* standard error of the mean.

^a,b^Values within the same row with different superscripts denote significance differences (*P* < 0.05).

^1^AO postbiotic fed at 3 g/calf/day and mixed with milk replacer.

### Heat stress altered metabolism of energy-yielding metabolites

**Figure 3:**
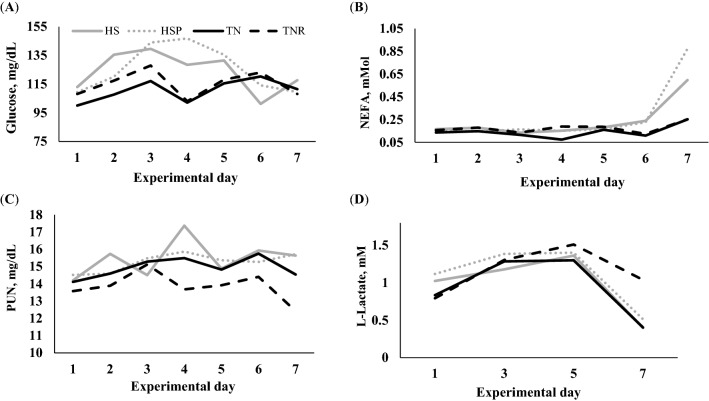
(**A**) HS and HSP treatments increased plasma glucose concentrations (treatment by day interaction; *P* < 0.005; SEM = 7.47 mg/dL; *TN* thermoneutral, *TNR* thermoneutral feed-restricted, *HS* heat stress, *HSP* heat stress and dietary supplementation with 3 g of AO postbiotic; n = 8 Holstein bull calves per treatment). (**B**) HS and HSP treatments increased plasma NEFA concentrations (treatment by day interaction; *P* < 0.033; SEM = 0.021 mM). (**C**) The TNR treatment decreased plasma PUN concentrations (13.8 mg/dL) relative to HS, HSP, and TN (*P* ≤ 0.007). (**D**) Plasma l-lactate concentrations were not affected by treatments (SEM = 0.07 mM).

Both HS and HSP calves experienced increased levels of circulating glucose, but this effect was influenced by day (treatment by day interaction *P* = 0.004; Fig. 3A). Compared with TN and TNR, the HS treatment increased plasma glucose concentrations on day 2 and 3 and tended to increase (*P* < 0.10) on day 4 and 5, whereas HSP treatment increased plasma glucose concentrations on day 3, 4, and 5. Notably, on day 4, HSP treatment promoted an even greater increase in plasma glucose concentration than HS (128.5 vs. 146.8 mg/dL; *P* = 0.027). There was a treatment by day interaction for plasma NEFA concentrations (*P* = 0.034; Fig. 3B) because HS and HSP increased circulating NEFA on day 6 and 7 compared with TN and TNR treatments. The HS, HSP, and TN treatments registered higher (*P* = 0.007) PUN concentrations than TNR (Fig. 3C); whereas plasma levels of l-lactate remained unaffected (Fig. 3D). Even though statistical differences in the entry rate of amino acids were not observed, HS, HSP and TNR treatments showed numerical differences relative to TN (Table 4). Furthermore, entry rates of several essential amino acids, most notably lysine, were numerically greater in HSP calves compared with HS counterparts (Table 4). Indeed, analysis of this difference showed that HS tended (*P* = 0.07) to decrease lysine entry rate by 167% compared with HSP. Our exploratory research suggests that a greater number of replications would likely increase the statistical power to declare such differences as significant. Additional results are shown under supplementary information (see Supplementary Tables).

**Table 4:** Effects of feed restriction, heat stress, and heat stress with supplementation of a postbiotic on relative entry rates of plasma amino acid (n = 4 per treatment).

Entry rate^2^	Treatment				SEM	*P*-value
	TN	TNR	HS	HSP^1^		
**Essential AA**						
Ile	1.00	0.21	0.50	0.43	0.21	0.27
Leu	1.00	0.45	0.31	0.52	0.24	0.32
His	1.00	0.83	1.03	1.65	0.48	0.68
Lys^3^	1.00	0.77	0.33	0.88	0.19	0.18
Met	1.00	0.43	0.58	0.43	0.27	0.51
Phe	1.00	0.40	0.26	0.53	0.26	0.34
Thr	1.00	1.05	0.80	1.40	0.47	0.82
Val	1.00	0.15	0.39	0.23	0.39	0.54
**Non-essential AA**						
Ala	1.00	3.40	4.50	13.50	5.60	0.39
Asx	1.00	0.66	0.66	0.35	0.29	0.56
Gly	1.00	0.95	0.61	0.89	0.17	0.50
Pro	1.00	1.03	0.59	1.01	0.29	0.71
Tyr	1.00	0.66	0.58	0.62	0.31	0.81
Glx	1.00	0.70	14.30	10.90	8.20	0.55
Ser	1.00	0.95	0.22	1.28	0.44	0.44

*TN* thermoneutral, *TNR* thermoneutral feed-restricted, *HS* heat stress, *HSP* heat stress-postbiotic, *SEM* standard error of the mean.

^1^AO postbiotic fed at 3 g/calf/day and mixed with milk replacer.

^2^Plasma AA entry rates in TNR, HS, and HSP were expressed relative to TN treatment.

^3^Plasma Lys entry rate tended (*P* = 0.07) to decrease more in HS compared with HSP calves.

## Discussion

In line with our experimental objectives, the temperature in the heat stress room mimicked a change that is typically observed in spring and summer dairy regions of the world^3,11^. Under this setting, and contrary to our projections, we found that HS calves showed similar feed intake compared to TN counterparts. Body temperature data confirm that animals housed under heat stress conditions experienced increased diurnal mean body, rectal, and skin temperature throughout the study. Furthermore, feed efficiency, total and partial energy efficiencies, diminished in HS compared with TN and TNR calves. These results were expected because the metabolic implications of the stress response include the reallocation of energy and nutrients to reestablish homeostasis which, in turn, reduces resources available to support other biological functions like growth^13^. The lack of heat stress effect in feed intake of HS calves agrees with previous work in cattle^3^. Feed intake of TNR and HSP animals did not differ and displayed a ~ 10% lower feed consumption compared with TN and HS counterparts. The decline in feed intake of HSP is within the range observed in previous reports in heat-stressed calves^13^. It is not clear, however, if this effect is a direct consequence of the administration of the postbiotic because in this study a treatment of thermoneutral calves consuming the postbiotic was not included. However, such a possibility is unlikely because in our previous study^11^ the same postbiotic did not affect feed intake in lactating dairy cows. Furthermore, HSP animals maintained partial energetic efficiency at similar levels compare to calves housed under thermoneutral conditions. Collectively, these findings demonstrate that our experimental model successfully induced heat stress in calves and allow speculating that the energy metabolism of heat-stressed calves might be improved by dietary supplementation with the AO postbiotic.

Compared with TN and TNR, HS and HSP calves showed elevated skin rump temperature at 0700 h suggesting that these animals maintained elevated skin temperature at nighttime. Heat stress triggers dynamic adaptive physiologic responses associated with a substantial flow of heat from the core to peripheral tissues. Typically, the temperature of the peripheral thermal compartment shows 0.5–6.0 °C^14,15^ less than core temperature. However, this thermal gradient can range from nearly zero to 6.0 °C or more depending on the severity of the thermal stress and the consequent vasomotor responses^16^. For example, heat stress triggered a 300% increase blood flow in peripheral tissues in mammals^6^. Thus, in our model, heat stress is associated with substantial changes in the core-to-peripheral tissue temperature gradient and distribution of body heat. Body heat distribution is mainly the result of two thermal compartments, namely the core and peripheral tissues. Mean-body temperature, defined as the mass-weighted average of core and skin temperatures, is thus a fundamental characterization of an animal’s thermal status. Mean-body temperature data confirmed that the heat load in heat stressed-calves increased from day 1 to 7. Our results showed that the mean body temperature peaked earlier (1500 h) than the rectal temperature (1900 h) indicating that the maximum thermal load was reached at 1500 h, about 7 h after the initiation of thermal stress. Metabolic heat production is relatively small in dairy bull calves which are typically consuming low-fiber diets relative to lactating dairy cows. The large surface area to mass ratio of calves may lead to increased absorption of heat from the environment, and this probably influenced body temperature in our study.

Our findings suggest that the heat stress-induced response reprogramed metabolism in multiple ways to support new metabolic priorities. First, both HS and HSP treatments might have changed carbohydrate metabolism by altering temporal patterns of glucose availability and the and magnitude of those changes, particularly on day 2 through 5. Second, HS and HSP triggered a dramatic increase of plasma NEFA concentrations on day 6 and 7. One possible interpretation is that secretion of catabolic stress hormones that coordinate energy metabolism increases and stimulates glycogenolysis, endogenous glucose production, and hepatic glucose output^17^. These long-lasting responses deplete storage of glycogen and, in turn, stimulate lipolysis of adipose to release NEFA and provide substrates for ATP production. Collectively, data presented here suggest that HS and HSP treatments increased availability and metabolism of carbohydrate. Upon utilization of glucose in circulation and stored, lipolysis and NEFA mobilization increased.

As predicted, exposing calves to heat stress increased small intestine permeability. This response was probably mediated by a reduction of the mucosal surface area, increased leak pathway of paracellular movement of water and nutrients, or both. These changes appeared to be located at the small but not the large intestine, as suggested by the results obtained from gut markers analysis. In agreement with our results, increased intestinal permeability has been also observed in other heat stress animal models^5,6^. HSP calves had similar intestinal permeability relative to TN and TNR calves suggesting that the postbiotic may have improved the barrier function of the intestine in these calves. Further to this point, HSP calves had lower fecal water content suggesting that this treatment may have improved water absorption, hence, gut functionality. The precise mechanism has not been addressed by the experimental design of this study, but future work should be designed to identify a mechanism of action to increase our understanding of the intestinal barrier function.

Serum amyloid A results for HS and HSP treatments and haptoglobin and lipocalin-2 results for the HSP treatment suggest a systemic proinflammatory state in these calves. These results agree with previous studies in ruminant and nonruminant animals for which heat stress increased markers of systemic inflammation^18–20^. However, our previous study in heat-stressed cows consuming AO postbiotic showed a quadratic reduction of serum amyloid A and lipopolysaccharides binding protein highlighting the effectiveness of 3 but not 6 g/day of postbiotic supplementation on markers of systemic inflammation^11^. Despite the lack of reduction of inflammatory markers, animal productivity improved in calves receiving the postbiotic. The latter agrees with beneficial effects of supplementing 3 and 6 g/day of AO postbiotic on productivity observed in previous work in heat-stressed lactating cows^11^.

In addition, systemic inflammatory responses via the actions of lipopolysaccharides and pro-inflammatory cytokines have been proposed and reported as a hallmark of heat-induced tissue injury when core temperature > 6 °C relative to control in heat stroke studies in rodents^6,7,16,19^. The increase in systemic inflammation may be a consequence of dysfunction intestinal permeability which is typically paralleled with increased translocation of luminal microorganisms and their products into the intestinal tissue and blood circulation as described during heat stroke in rodents. In addition to lumen-associated inflammation, an increase of the oxidative status can lead to systemic inflammation^5,6^.

Results indicated that plasma concentration of zonulin increased in TNR animals only. These results are puzzling because increased concentrations of zonulin have been reported as part of a long-term response associated with inflammatory processes^21^.

In summary, our findings indicated that the heat stress treatment led to reduced nutrient-use efficiency and increased body temperature and systemic inflammation. Results indicated that the postbiotic improved energy-use efficiency, water absorption, and the intestinal permeability but did not reduce heat stress-mediated rise in markers of systemic inflammation.

## Materials and methods

The experiment conducted herein was approved by the Institutional Animal Care and Use Committee of the University of Tennessee (protocol no. 2655-0219). All experimental procedures were performed in accordance with the animal ethics approval and regulations. The study was carried out in compliance with the ARRIVE guidelines.

### Experimental design

A total of thirty-two (1- to 2-week-old) bull calves were obtained from a commercial operation and raised following industry standard recommendations. Calves [body weight (BW) = 121 ± 2.2 kg; 12 ± 1 weeks of age; mean ± SD] were housed in individual pens in climate-controlled rooms (19.8 ± 0.8 °C constant ambient temperature) 3 day prior to the study at the East Tennessee Research and Education Center—Johnson Animal Research and Teaching Unit at the University of Tennessee-Knoxville^14,22^. Each room accommodated 8 pens so that the study was conducted in 2 cohorts of 16 calves each. Calves were housed at either thermoneutral (TN; constant 19.5 °C ambient temperature) or heat stress (HS; diurnal maximal ambient temperature of 37.8 °C) for 7 days. Diurnal HS climate resulted in 12 h/day of heat stress from day 1 through 7^22^ (Supplementary Figures). Commercial milk replacer was fed in bottles to each individual animal once daily at 0500 h at 340 g following industry recommendations^23^. Water was offered ad libitum four times daily at 0500, 1200, 1700, and 2000 h. Calves did not show signs of health issues prior and during the course of the study.

### Treatments

Calves were randomly assigned to 1 of 4 treatment groups (n = 8 calves/treatment). Treatments were (1) TN conditions fed ad libitum starter (TN), (2) HS conditions fed ad libitum starter (HS), 3) HS supplemented with 3.0 g/calf/day of AO postbiotic in milk replacer (HSP; Biozyme, Inc., St. Joseph, MO), and TN with ~ 8% restriction of starter consumption (TNR). The postbiotic was mixed in each bottle thoroughly with milk replacer to ensure consumption and post-ruminal delivery. The postbiotic was administered 13 days prior to imposing heat stress^11^ and continue until the end of the study on day 7. Full consumption of milk replacer was confirmed on each individual calf prior and during allocation to temperature-controlled rooms. Calf starter was offered four times daily at 0500, 1200, 1700, and 2000 h to allow for 5–10% refusal (i.e. ad libitum) in the TN, HS, and HSP treatments. The feed intake restriction imposed in TNR calves was based on feed intake data reported on heat-stressed growing dairy cattle^13^. Body weights were measured prior to the administration of the postbiotic and used as covariate in the statistical analysis (mean group body weight was 98, 102, 101, and 101 kg/animal for HS, HSP, TN, and TNR, respectively).

### Thermal load assessment

The temperature and relative humidity in the rooms were monitored on day 1 to 7 every 10 min using HOBO U23 Pro v2 (Onset Computer Corp., Bourne, MA; accuracy ± 0.21 °C and 2.5% relative humidity) as previously used^14,24^. Each calf’s thermal response was evaluated for four times daily at 0700, 1100, 1500, and 1900 h using rectal temperature (RT; GLA M700 digital thermometer; accuracy ± 0.1 °C), skin temperatures (ST) at a clean shaven 10 cm × 10 cm patch on the rump at ~ 15 cm in distance (FLIR imaging gun; accuracy ± 1.5 °C), and respiration rates by counting flank movements for 15 s and reported as breaths/min. Additional RT data collected on HS and HSP calves was obtained every 60 min from 0700 to 2000 h. Mean body temperatures (MBT) were calculated using RT and ST in the following equation^25^: MBT = (RT × 0.70) + (ST × 0.30).

### Performance measurements

Body weight was measured on day 1 and 7, and consumption of water, milk replacer, and starter was recorded daily on day 1 through 7. All calves consumed the totality of milk replacer offered and feed intake was calculated by adding the amount consumed of milk replacer and starter on a DM basis. Feed to gain ratio was calculated as kg of total intake on DM basis/kg of BW gain. Total energetic efficiency was calculated as the gross energy gain/metabolizable energy intake^26^, and partial energetic efficiency was calculated as gross energy gain/the difference between metabolizable energy intake and net energy maintenance^27^. Samples were taken of the milk replacer and the pelleted starter to analyze nutrient contents (Supplementary Tables). Samples of rectum content collected on day 3, 4, 5, 6, and 7 were used to determine water content in feces.

### Analysis of plasma proteins and metabolites

Individual blood samples were collected at 0700 h daily on day 1 to 7 by jugular venipuncture in sodium heparin tubes and separated for plasma collection at 1200×*g* for 10 min at 4 °C within 30 min and stored at − 80 °C. Plasma acute phase proteins were analyzed using enzyme-linked immunosorbent assays (bovine haptoglobin: Immunology Consultants Laboratory, Inc., Portland, Oregon; multispecies serum amyloid A : Tridelta Development, Maynooth, County Kildare, Ireland^18^) on day 1, 3, 5, and 7. Bovine Lipocalin-2 was detected according to manufacturer protocol (MyBioSource, Inc., CA; Catalog N MBS018977) on day 1, 3, 5, and 7. The biochemical technique is based on Lipocalin-2 antibody-Lipocalin-2 antigen interactions (immunosorbency) and a colorimetric detection system to detect Lipocalin-2 antigen targets in samples. Bovine zonulin was detected using enzyme-linked immunosorbent assay kit according to manufacturer protocol (Haptoglobin Precursor; Antibodies-online Inc., PA; Catalog No. ABIN992457) on day 1, 3, 5, and 7. Plasma glucose, urea-N, and NEFA concentrations were determined on day 1 through 7. Glucose and urea-N concentrations were determined using commercially available enzymatic assays (Sigma-Aldrich, St. Louis, MO). Plasma NEFA concentrations were determined using commercial assay kit (Wako Diagnostics, Mountain View, CA). Plasma l-lactate concentrations were determined using a commercial assay kit (BioAssay Systems, EnzyChrom (ECLC-100), # CA09A28) on day 1, 3, 5, and 7. Concentration of metabolites were determined using a microplate spectrophotometer (BioTek Synergy H1 Multi-Mode Reader; Winooski, VT). Intra-assay and inter-assay coefficients of variation showed a range of 1.0 to 16.7%.

### Analysis of blood gases

Whole blood samples collected on day 1 and 7 were used to conduct blood gas analysis using i-STAT analyzer according to protocol provided by manufacture (Abbott Point of Care Inc., Princeton, NJ; Supplementary Tables).

### Intestinal permeability

Small intestine permeability was assessed by adding lactulose (0.50 g/kg BW) and mannitol (0.10 g/kg BW) in milk replacer at 0500 h on day 7 (Sigma-Aldrich, St. Louis, MO^28^). Blood samples collected at 0700 h were used to harvest plasma then stored at − 80 °C until analysis. Plasma was submitted to a commercial lab to determine concentrations of the synthetic sugars using high performance liquid-chromatography coupled with mass spectrometry (University of North Texas, Denton, TX^29^).

### Amino acid absorption

An exploratory analysis was conducted on 12 calves randomly selected (n = 4/treatment). Two catheters were placed into ipsilateral jugulars on day 6^14^. On day 7, a sterile stable isotope-labeled AA mixture (0.10 g of ^13^C-labeled AA, 4.4 mg of ^13^C-labeled L-Met, and 5.7 mg of ^13^C-labeled L-His-HCl-H_2_O dissolved in 120 mL of saline) was infused into one of the catheters over 8 h at a constant rate of 1.0 mL/min using medical peristaltic pumps (Plum XL IV; Abott-Lifecare, San Antonio, TX). Twelve blood samples (5 mL) per calf were taken over the entire infusion period from the other jugular catheter into Na-EDTA tubes. Plasma was collected at 1200 × *g* for 10 min at 4 °C within 60 min and stored at − 80 °C. Plasma samples were prepared for ion ratio mass spectrometry analysis to determine the intestinal entry rate of individual AA as previously described^30^.

### Amino acid model descriptions and parameter estimation

Briefly, a 4-pool dynamic model was constructed and used to estimate plasma amino acid (AA) entry rates and AA turnover rates between the fast and slow pool as previously described^30^. The fast pool represents blood, interstitial, and cytoplasmic free AA, which was assumed as 14.9% of BW. The slow pool represents protein-bound AA and was calculated using the assumption that body protein is 18.8% of BW^31^. The AA composition of the fast and slow pools were set based on plasma and muscle AA concentrations as previously reported^32^. Model predictions of isotope ratios in the fast pool were fitted to the observed plasma AA isotope ratios for each AA within the infusion by maximizing a log-likelihood function using the Nelder-Mead optimization algorithm^33^. To adjust AA intake among individual animals, AA relative bioavailability was calculated by dividing plasma AA entry rate by AA intake. All modeling work was conducted in R (version 3.5.1^34^).

### Statistical analysis

Data were analyzed using a mixed model in SAS version 9.4 (SAS Institute Inc., Cary, NC). Data were analyzed for homoscedasticity and normality of residuals. Body weight data collected prior to the beginning of the postbiotic feeding were included as a covariate adjustment in the model. Best-fit models were determined using backwards manual selection, specifically taking low Akaike information criterion (AIC) into consideration. All models included the overall mean, the fixed effect of treatment, the fixed effect of replica, the random effect of calf, the covariate effects, and the random error. A repeated measure was included in the model for non-random and consecutive measurements taken over time (h or day). Repeated measures procedure was used to determine overall differences related to treatments and time and treatment by time interactions. Covariate analysis was included in the model if statistically significant (*P* ≤ 0.05). Thermoregulatory responses related to changes in ambient temperature and time were characterized using treatment replica, time effects, and all interactions in the model. Significant differences were declared at *P* ≤ 0.05, and trends were declared at 0.05 < *P* ≤ 0.10. All results are reported as least squares means or slopes ± standard error of the mean. Models to characterize thermoregulatory responses with treatments were also tested to determine if ambient and rectal temperatures captured the information in both variables. Rectal temperature was characterized using treatment and ambient temperature regression effects. Respiration rate was characterized using treatment, ambient temperature, and rectal temperature regression effects.

## Supplementary Information

Supplementary Information.

## Data Availability

All the data supporting these findings are present within the manuscript.
