# Supplementary material for: Physiological responses of Holstein calves to heat stress and dietary supplementation with a postbiotic from Aspergillus oryzae
Source: Sci Rep. 2022 Jan 28;12:1587. doi: 10.1038/s41598-022-05505-3 (PMC8799720; doi:10.1038/s41598-022-05505-3)
Supplement: Supplementary file 1 — Supplementary Information. [file 41598_2022_5505_MOESM1_ESM.pdf]

Supplementary Information

Physiological responses of Holstein calves to heat stress and dietary supplementation with a postbiotic from *Aspergillus oryzae*

A. G. Ríus[1*], J. D. Kaufman[1], M. M. Li[2], and M.D. Hanigan[2], I. R. Ipharraguerre[3*]

[1]Department of Animal Science, University of Tennessee, Knoxville, TN 37996

[2]Department of Dairy Science, Virginia Tech, Blacksburg, VA 24061

[3]Institute of Human Nutrition and Food Science, University of Kiel, Germany

Agustín G. Ríus

2506 River Drive

235 Brehm Animal Science Building

Knoxville, Tennessee 37996

Phone: (865) 974-3150

Fax: (865) 974-7297

Email: arius@utk.edu

Supplementary Results

Mean skin temperature in the rump was elevated in HS and HSP compared with TN and TNR animals at 0700, 1100, 1500, and 1900 h (treatment by h interaction; $P < 0.001$; Supplementary Figure S2). These results suggest that the HS and HSP animals did not recover fully from the heat load and maintained elevated skin temperature overnight. The increase in ambient temperature resulted in an increase of mean body temperature for HS and HSP compared with TN and TNR calves at 0700, 1100, 1500, and 1900 h (treatment by h interaction; $P < 0.001$; Supplementary Figure S2). Mean body temperature at 1100, 1500, and 1900 h were on average 2.0, 2.4, and 2.0°C greater in HS and HSP compared with TN and TNR calves. The TNR calves increased ($P < 0.008$) mean body temperature (0.2°C) at 1100 h and tended to decrease ($P = 0.08$) mean body temperature (0.2°C) at 1900 h compared with the TN calves. Mean body temperature on HSP animals declined compared with HS at 1900 h ($P \leq 0.01$).

Supplemental Figures and Tables.

Supplementary Figure 1. (A) Daily pattern of ambient temperature in thermoneutral (TN) and heat stress (HS) rooms. Ambient temperature remained constant at an average of 19.1°C for the TN room whereas temperature ranged from 19.1 to 37.8°C from 0800 to 1900 h in the HS room (each point shows 10-minute temperature average). (**B**) Aritmetic mean of ambient temperature and relative humidity in heat stress room on days 1 to 7.

(**A**)

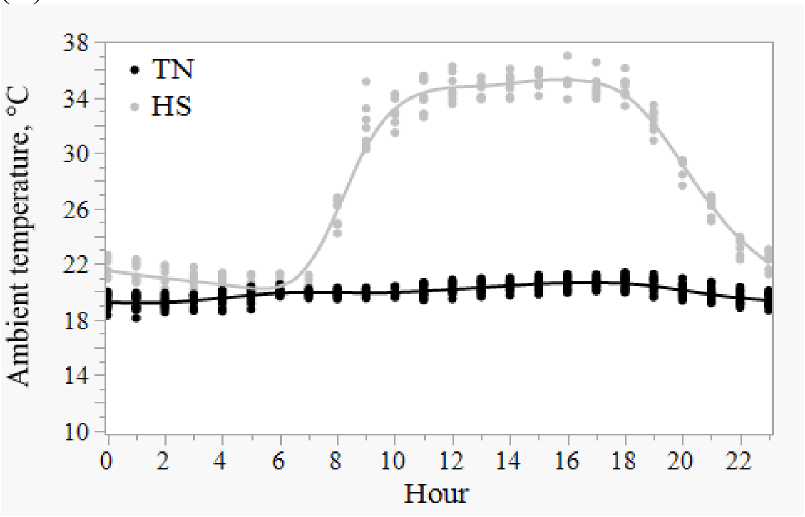

(**B**)

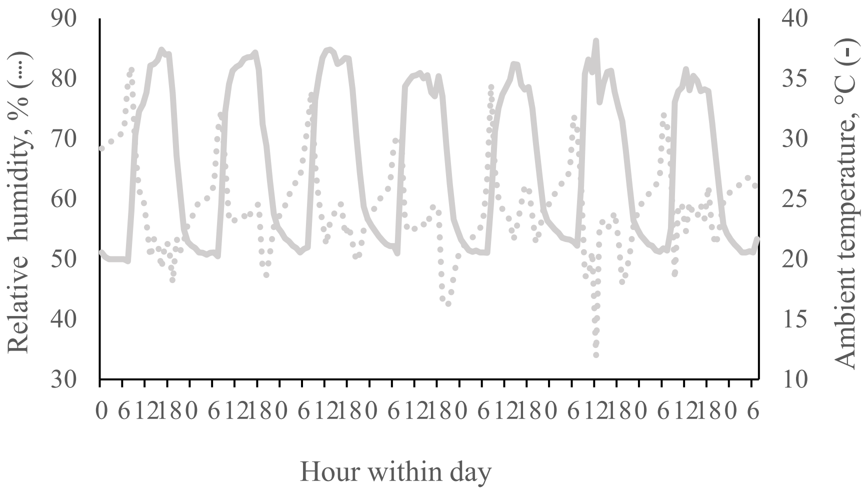

Supplementary Figure 2. (A) Mean rump skin temperature of Holstein bull calves (treatment by h interaction, *P* < 0.001; TN = thermoneutral; TNR = thermoneutral feed-restricted; HS = heat stress; and HSP = heat stress and dietary supplementation with 3 g of AO postbiotic; n = 8 Holstein bull calves per treatment). P ≤ 0.01 denoted by * (compared with TN), # (compared with TNR), or ∞ (compared with HS). Mean rump skin temperature in HS and HSP treatments differ compared with TNR and TN. Compared with TN, TNR treatment increases (1100) and reduces skin temperature (1900). (**B**) Mean body temperature [(rectal temperature × 0.70) + (skin temperature × 0.30)] of calves (treatment by h interaction, *P* < 0.001). Results show least squares means + standard error of the mean. (**C**) Mean rectal temperature of HS and HSP treatments differ compared with TN and TNR.

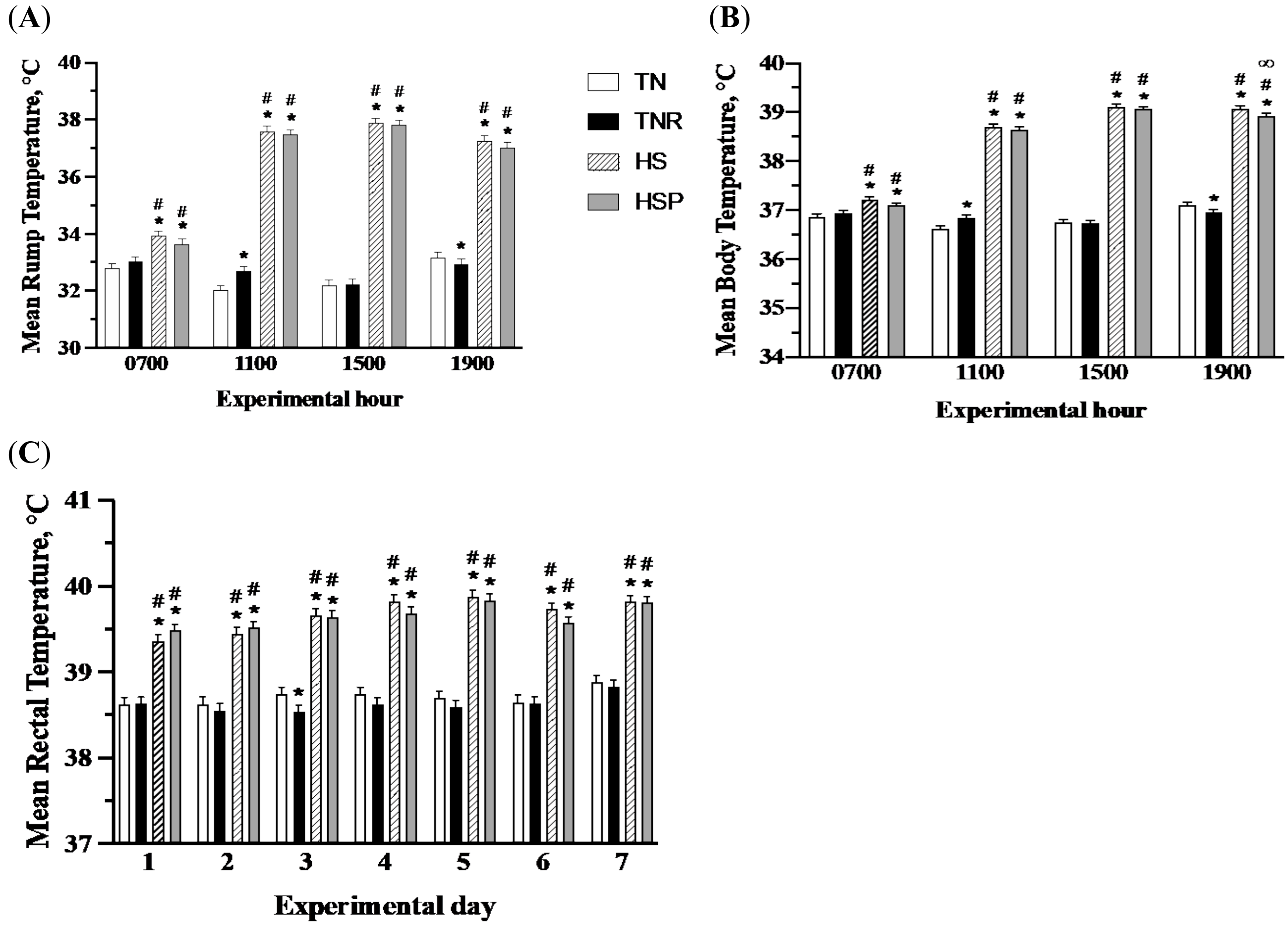

Supplementary Figure 3. Relationship between respiration rate (breath per minute, bpm) and ambient temperature of Holstein bull calves (n = 8 per treatment) exposed to thermoneutral environment (TN), thermoneutral feed-restricted (TNR), heat stress (HS), and heat stress and dietary supplementation with 3 g of AO postbiotic (HSP). Dark gray shaded area shows 95% mean confidence interval and light gray shaded area represent 95% prediction limits. Calves in HS and HSP treatments showed a linear relationship for ambient temperature ($P < 0.0001$) with respiration rate = 39.1 - 4.2x ($R^2 = 0.59$) and 37.1 - 4.1x ($R^2 = 0.65$), respectively.

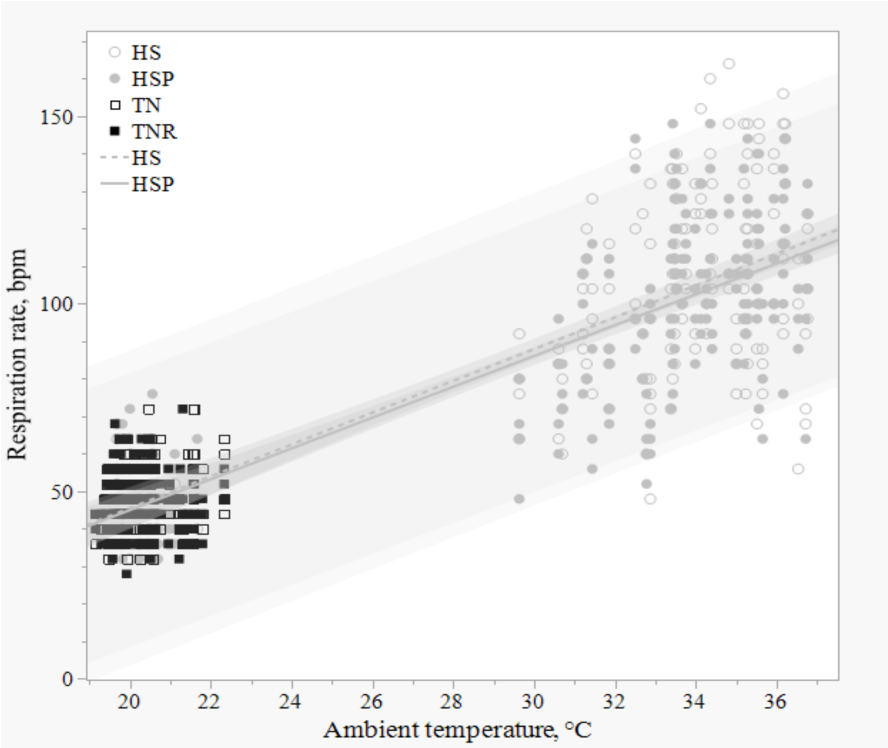

Supplementary Figure 4. Fecal water content (treatment by experimental day interaction, *P* = 0.991, SEM = 0.32%; n = 8 Holstein bull calves per treatment; TN = thermoneutral; TNR = thermoneutral feed-restricted; HS = heat stress; and HSP = heat stress and dietary supplementation with 3 g of AO postbiotic).

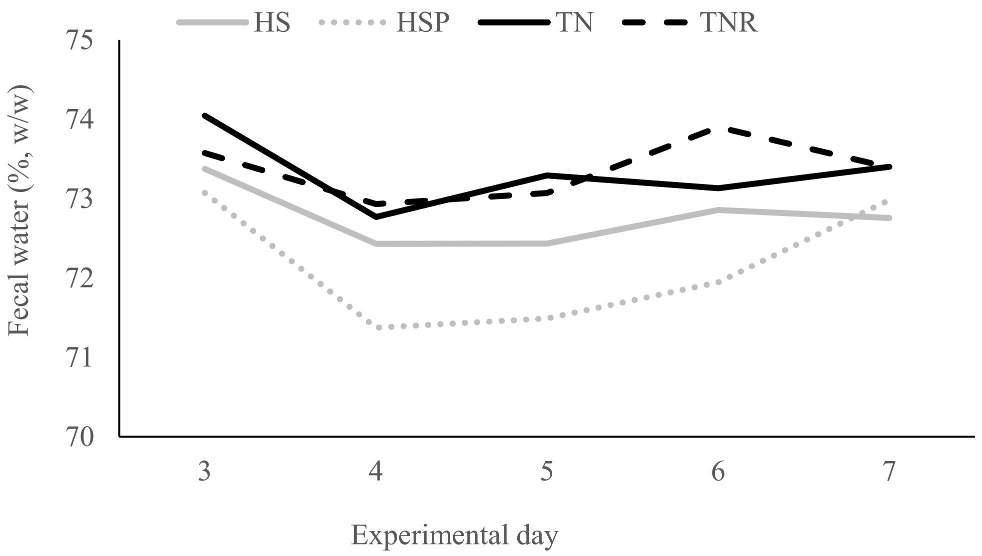

Supplementary Figure 5. (A) Plasma haptoglobin (treatment by experimental day interaction, *P* = 0.895, SEM = 0.042 µg/ml; n = 8 Holstein bull calves per treatment; TN = thermoneutral; TNR = thermoneutral feed-restricted; HS = heat stress; and HSP = heat stress and dietary supplementation with 3 g of AO postbiotic). **(B)** Plasma serum amylose A (treatment by experimental day interaction, *P* = 0. 878, SEM = 0.221 µg/ml). **(C)** Plasma zonulin (treatment by experimental day interaction, *P* = 0. 716, SEM = 9.8 pg/mL). **(D)** Plasma lipocalin-2 (treatment by experimental day interaction, *P* = 0. 505, SEM = 48.9 ng/ml).

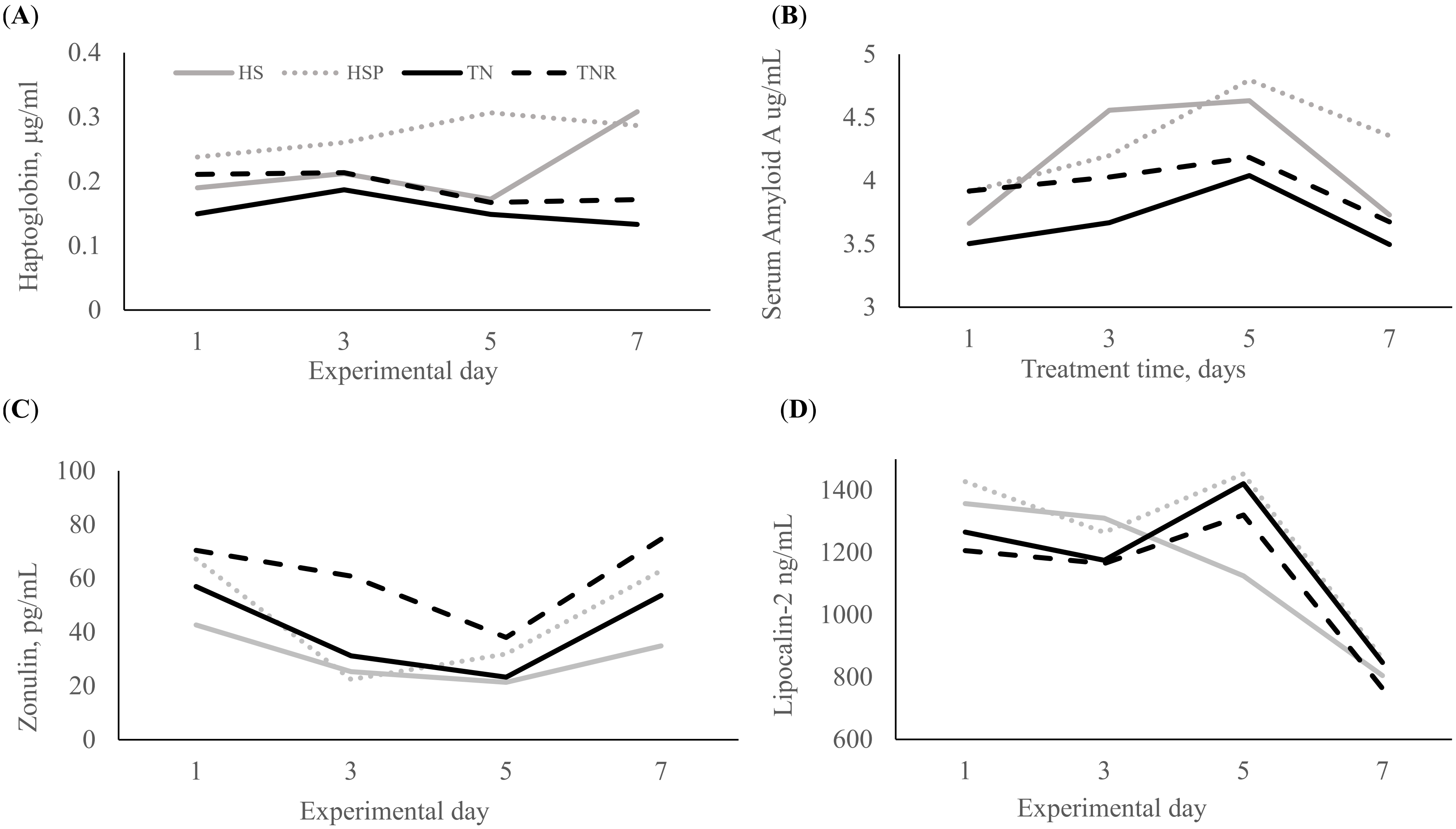

Supplemental Table 1. Blood parameters of Holstein bull calves (n = 8 calves per treatment; TN = thermoneutral; TNR = thermoneutral feed-restricted; HS = heat stress; and HSP = heat stress and dietary supplementation with 3 g of AO postbiotic).

Parameter	TN	TNR	HS	HSP	SEM	*P*-value
pH	7.4	7.4	7.4	7.4	0.01	0.21
pCO_2, mmHg	44.8^{ab}	46.3^{a}	41.0^{bc}	40.1^{c}	1.05	0.05
pO_2, mmHg	32.6	32.6	34.5	35.5	2.18	0.75
Total CO_2, mmol/l	29.1	29.9	28.7	28.5	1.42	0.71
HCO_3, mmol/l	27.2	28.2	27.8	27.6	1.45	0.83
Base excess, mmol/l	2.9	3.6	3.0	3.3	1.52	0.93
Saturated O_2, %	60.6	61.5	66.8	68.7	4.00	0.51
Hemoglobin, g/dl	8.2	8.1	8.1	8.4	0.31	0.89
Hematocrit, % PCV	24.1	23.8	23.8	24.7	0.89	0.89
Sodium, mmol/l	139.5	139.5	138.7	137.8	0.52	0.25
Potassium, mmol/l	3.9	3.8	4.0	4.2	0.08	0.11
Ionized Calcium, mmol/l	1.4	1.3	1.4	1.4	0.02	0.19

pCO_2. Partial pressure of carbon oxide; pO_2, partial pressure of oxygen; HCO_3, bicarbonate.
[a,b] Values within the same row with different superscripts denote significance differences ($P <$ 0.05). HS and HSP decreased ($P = 0.05$) pCO_2 concentration compared with TNR treatment.

Supplemental Table 2. Relative turnover rate of amino acids between fast and slow pools in Holstein bull calves (n = 4 calves per treatment; TN = thermoneutral; TNR = thermoneutral feed-restricted; HS = heat stress; and HSP = heat stress and dietary supplementation with 3 g of AO postbiotic).

	Treatment					
Turnover rate	TN	TNR	HS	HSP	SEM	*P*-value
Essential AA						
Ile	1.00	0.50	0.71	0.32	0.36	0.64
Leu	1.00	0.48	1.18	0.45	0.52	0.65
His	1.00	2.01	0.68	0.42	1.26	0.81
Lys	1.00	1.31	1.81	0.68	0.68	0.64
Met	1.00	0.59	1.59	1.97	1.00	0.79
Phe	1.00	0.41	1.08	0.25	0.41	0.45
Thr	1.00	0.46	0.59	0.41	0.22	0.35
Val	1.00	0.19	0.28	0.26	0.21	0.12
Non-essential AA						
Ala	1.00	0.20	0.35	0.28	0.20	0.13
Asx	1.00	0.63	0.74	0.54	0.51	0.94
Glx	1.00	0.63	0.25	0.41	0.20	0.18
Gly	1.00	1.89	1.89	1.89	1.00	0.89
Pro	1.00	0.34	0.72	0.25	0.25	0.27
Ser	1.00	0.53	0.86	0.42	0.32	0.54
Tyr	1.00	0.54	1.85	0.31	1.00	0.66

Supplemental Table 3. Relative bioavailability of amino acid in Holstein bull calves (n = 4 calves per treatment; TN = thermoneutral; TNR = thermoneutral feed-restricted; HS = heat stress; and HSP = heat stress and dietary supplementation with 3 g of AO postbiotic).

	Treatment					
Bioavailability	TN	TNR	HS	HSP	SEM	*P*-value
Essential AA						
Ile	1.00	0.26	0.57	0.55	0.25	0.33
Leu	1.00	0.52	0.30	0.57	0.26	0.42
His	1.00	0.92	1.10	2.10	0.49	0.41
Lys	1.00	0.90	0.33	1.04	0.21	0.14
Met	1.00	0.51	0.58	0.51	0.31	0.71
Phe	1.00	0.49	0.31	0.61	0.29	0.50
Thr	1.00	1.28	0.86	1.68	0.53	0.69
Val	1.00	0.21	0.46	0.30	0.44	0.66
Non-essential AA						
Ala	1.00	14.00	15.00	27.80	17.20	0.33
Asx	1.00	0.79	0.65	0.41	0.33	0.69
Glx	1.00	1.20	21.60	16.80	12.80	0.56
Gly	1.00	1.04	0.61	1.05	0.19	0.34
Pro	1.00	1.17	0.59	1.17	0.37	0.61
Ser	1.00	1.18	0.25	1.46	0.49	0.37
Tyr	1.00	0.80	0.58	0.70	0.34	0.86

Supplemental Table 4. Chemical composition of milk replacer and starter consumed by Holstein bull calves (% of DM or stated otherwise).

Items	Milk replacer	Starter
Crude protein	26	18
Crude fat	20	2
Crude fiber	0.15	15
Acid detergent fiber	-	21
Calcium	0.75	1.2
Phosphorus	0.7	0.55
Salt	-	0.55
Potassium	-	0.8
Selenium (ppm)	-	0.3
Vitamin A (IU/kg of DM)	6,820	3,800
Vitamin D-3 (IU/kg of DM)	2,280	800
Vitamin E (IU/kg of DM)	45	23

Supplemental Table 5. Amino acid composition (% of DM) of starter and milk replacer.

	Starter	Milk replacer
Essential AA		
His	0.43	0.58
Ile	0.54	1.6
Leu	1.32	2.79
Lys	0.69	2.47
Met	0.28	0.56
Phe	0.77	1.04
Val	0.78	1.68
Thr	0.56	0.76
Nonessential AA		
Ala	0.85	1.19
Tyr	0.53	0.97
Pro	1.03	1.8
Glx	3	4.67
Asx	1.29	2.67
Gly	0.8	0.5
Ser	0.79	1.39
